# Prohexadione-calcium improves grape quality by regulating endogenous hormones, sugar and acid metabolism and related enzyme activities in grape berries

**DOI:** 10.1186/s12870-024-04803-4

**Published:** 2024-02-19

**Authors:** Dou Li, Jiangshan Yang, Zibo Dai, Yajuan Chen, Zhang Shao, Chunheng Wang, Xin Jin, Yuhang Wang, Lidan Feng

**Affiliations:** 1 College of Horticulture, Gansu Agricultural University, Lanzhou, 730070 China; 2 Research and Development Center of Wine Industry in Gansu Province, Lanzhou, 730070 China

**Keywords:** Wine grapes, Pro-ca, Sugar and acid metabolism, Endogenous hormones, Aroma substances

## Abstract

Prohexadione-Calcium (Pro-Ca) plays key roles in improving fruit quality and yield by regulating various aspects of plant growth. However, the effects of how Pro-Ca regulates the regulation of sugar and acid balance and its impact on the production of volatile aroma substances during fruit growth and development are poorly understood. In this study, the Pro-Ca solutions developed at concentrations of 200, 400, 600 and 800 mg·L^-1^ were sprayed on the entire “Chardonnay” grape tree 22, 42, 62 and 82 days after initial flowering. The values of endogenous hormones, sugar and acid content, enzyme activities and flavor content were then measured in grapes 45, 65, 85 and 105 days (ripeness stage) after the initial flowering. The results showed that Pro-Ca had significant effects on fruits during development, including reducing ABA content, increasing ZT, GA_3_ and IAA levels, promoting fruit ripening and enhancing enzymes, which are involved in sugar and acid synthesis. Consequently, these effects led to an increase in sugar and acid content in the berries. Particularly during the ripening phase, the application of 600 mg L^-1^ Pro-Ca resulted in an increase in soluble sugar content of 11.28% and a significant increase in citric acid and malic acid content of 97.80% and 68.86%, respectively. Additionally, Pro-Ca treatment enhanced both the variety and quantity of aroma compounds present in the berries, with the 600 mg·L^-1^ Pro-Ca treatment showcasing the most favorable impact on volatile aroma compounds in ‘Chardonnay’ grapes. The levels of aldehydes, esters, alcohols, phenols, acids, ketones, and terpenes were significantly higher under the 600 mg·L^-1^ Pro-Ca treatment compared to those of control with 51.46 − 423.85% increase. In conclusion, Pro-Ca can regulate the content of endogenous hormones and the activities of enzymes related to sugar and acid metabolism in fruit, thereby increasing the content of soluble sugar and organic acid in fruit and the diversity and concentration of fruit aroma substances. Among them, foliar spraying 600 mg · L^-1^ Pro-Ca has the best effect. In the future, we need to further understand the molecular mechanism of Pro-Ca in grape fruit to lay a solid foundation for quality improvement breeding.

## Introduction

The taste of fruits is determined by the combination of basic metabolites such as sugar, organic acid and volatile compounds. Together, these components influence the overall quality of the fruit [1].In wine grapes, the delicate balance between sugars and organic acids plays a fundamental role in the exceptional quality and distinct flavor of wine, which are mainly regulated by sucrose metabolism and the tricarboxylic acid cycle [2]. In addition, volatile aroma, as an important secondary metabolite in fruits, also plays a vital role in fruit flavor [3]. Recently, the study of volatile fruit aromas has gained popularity as detection and analysis techniques have advanced [4]. Currently, various methods such as soil improvement [5], pruning techniques [6], foliar micro-fertilization [7], and the application of growth regulators [8] are primarily utilized to increase the accumulation of sugar and organic acids in grape fruits, thereby enhancing fruit quality and flavor. With the improvement of people’s living standards and the emphasis on health, there is a rapidly increasing demand for quality fruit and vegetables [9]. Thus, how to efficiently improve the quality of grapes fruit has been a key area of research.

Prohexadione calcium (Pro-Ca) is an industrially synthesized plant growth regulator that is low-toxic and environmentally friendly [10]. Pro-Ca could promote plant development, improve fruiting rate and effectively control plant overgrowth, improving both yield and quality when applied to plant leaves [11]. In addition, new research has shown that Pro-Ca treatment also affects sugar-acid metabolism and aroma components in fruits, thereby affecting their taste and sensory properties. Studies have shown that the use of Pro-Ca can effectively regulate the concentration of important sugar acids such as malic acid and tartaric acid in fruit tissue [12]. In particular, Pro-Ca treatment has been shown to increase the accumulation of malic acid, which contributes significantly to fruit acidity and flavor improvement [12]. This change in sugar-acid composition caused by Pro-Ca treatment can have a significant impact on the overall flavor and quality of the fruit [13]. Furthermore, Pro-Ca treatment enhances fruit aroma by upregulating the biosynthesis of volatile compounds [14, 15]. Therefore, Pro-Ca treatment promises to be a valuable tool for manipulating sugar-acid metabolism and improving aroma profiles in fruits, ultimately leading to improved fruit quality and higher consumer satisfaction.

Previous studies have shown that Pro-Ca regulates plant growth by inhibiting gibberellin synthesis in plants [16]. However, there is limited research on the effects of exogenous Pro-Ca on the dynamic changes of endogenous hormones as well as fruit quality and flavor during grape growth and development. Therefore, in this study, Pro-Ca effervescent granules were used to spray the entire “Chardonnay” grape tree. The aim of the study was to analyze the influence on endogenous hormones, sugar and acid components, the activities of enzymes related to sugar and acid metabolism and the content of aromatic substances in different growth stages of grapes. This study of the effects of Pro-Ca on dynamic changes in sugar and acid metabolism and on the flavor quality of grapes is intended to provide a theoretical basis for improving the quality of grapes.

## Materials and methods

### Test materials

Plant materials: The 11-year-old ‘Chardonnay’ wine grape variety was selected as the test material. Only plants exhibiting consistent growth without pest or disease infestations were chosen. The grape plants were arranged in rows with a spacing of 0.75 m × 1.5 m, and a single-arm hedge frame was set up in a north-south direction. From April to October 2022, different concentrations of Pro-Ca were sprayed on the entire grape plant within the vineyard at Gansu Agricultural University.

Test reagent: The test reagent used in this study was Shibida brand Pro-Ca effervescent granules, which were produced by Anyang Quanfeng Biological Technology Co., Ltd.

The general situation of the test site is as follows: The site is located at coordinates N 36°5’ -37°10’, E 103°34’ -103°47’, with an elevation of approximately 1,517 m above sea level. It falls within the temperate climate zone and exhibits specific climatic characteristics including abundant sunlight, low precipitation, high evaporation, arid climate, soil drought, and an annual precipitation of 349.90 mm. The site experiences an annual evaporation of 1,664.00 mm and receives approximately 2,476 h of sunshine per year.

### Treatment and experimental designinary test

A randomized block design was employed for the experiment. The study consisted of five treatments: a control (0 mg·L^-1^ Pro-Ca), 200 mg·L^-1^ Pro-Ca, 400 mg·L^-1^ Pro-Ca, 600 mg·L^-1^ Pro-Ca, and 800 mg·L^-1^ Pro-Ca. Each treatment was replicated three times. Five plants displaying consistent growth and free from pests and diseases were selected within each plot. The whole plant was sprayed at 22, 42, 62 and 82 days (d) after the initial flowering, so that the leaves were full of water droplets, and labeling uniformly growing bunches after fruit set.

### Determination items and methods

Fruits were sampled at 45, 65, 85, and 105 d after the initial flowering, respectively, for a total of 4 times. The samples were fully frozen in liquid nitrogen and stored in an ultra-low temperature refrigerator at -80 ℃ for later use.

### Determination of endogenous hormones in fruits

The endogenous hormone contents of gibberellin (GA_3_), indole acetic acid (IAA), kinetin (KT), and abscisic acid (ABA) were determined using high-performance liquid chromatography. Following the method by Zhuo-heng CHI et al. [17], 2 g of grape pulp was rapidly ground into powder using liquid nitrogen. The powder was extracted three times with 10 mL of 80% chromatographic methanol (prepared with ultrapure water) into a 10 mL centrifuge tube. It was then refrigerated at 4 °C for 24 h to allow extraction. After centrifugation at 8000 rpm for 10 min, the supernatant was concentrated using a rotary evaporator at 38 °C to remove methanol, resulting in approximately 1 mL of concentrated solution. The walls of the evaporation bottle were washed with 50% chromatographic methanol, and the volume was finally adjusted to 1.5 mL. The concentrated solution was extracted using a disposable needle tube, filtered with a 0.22 μm organic membrane, and placed in a 1.5 mL sample bottle. The sample bottle was stored in a dark ice box and repeated three times. The contents of ZT, IAA, GA_3_, and ABA were determined using the following chromatographic conditions: Symmetry C18 column (4.6 mm × 250 mm, 5 μm); mobile phase of methanol 0.1% phosphoric acid (1:9, v/v); flow rate of 1.0 mL·min^-1^; detection wavelength at 254 nm; column temperature at 30 ℃; and an injection volume of 10 µL.

### Determination of soluble sugars, sugar components, and sucrose metabolism-related enzyme activities in fruits

The total soluble sugar content was determined using the anthrone method [18], The grape pulp was rapidly ground into powder with liquid nitrogen, 50 mg of dried-ground sample was added into a 10 mL centrifuge tube and added 5 mL of 80% ethanol. A glass ball was placed on top of the tube and kept in a water bath at 80–85 °C for 30 min. Centrifuged at 3,000 rpm for 10 min and decanted into a 50 mL volumetric flask, kept the residue in centrifuge tube, and repeat the extraction three times. The supernatant in 50 mL volumetric flask was then filled up by 80% ethanol. This extract was used for total soluble sugar content. For total soluble sugar analysis, 0.5 mL of soluble sugar extract and 4.5 mL of 80% ethanol were added into a test tube. Put the sample tubes into an ice bath and slowly added 10 mL of anthrone reagent to the tubes, and then place it in a boiling water bath for exactly 7.5 min and then immediately cooled in an ice bath. After cooling, the absorbance at 630 nm in 1 h was measured.

The sugar components were analyzed through high-performance liquid chromatography. The grape pulp was ground with liquid nitrogen, and 0.5 g of it was weighed. Next, 5 mL of 80% ethanol was added to the grape pulp, which was then ultrasonically extracted at 35 °C for 20 min. After centrifuging at 12,000 rpm for 15 min, the extraction process was repeated twice. On each occasion, 2 mL of 80% ethanol was added, and the resulting supernatant was combined to obtain a total volume of 10 mL. The solution was dried in a vacuum centrifugal concentrator at 60 °C. Subsequently, 1 mL of acetonitrile was redissolved with 1 mL of ultrapure water, filtered through a 0.22 μm organic phase microporous membrane, and transferred into a sample bottle for testing. Fructose, glucose and sucrose standards were used to prepare standard solutions of different concentrations, and then the content of sugar components was determined using the Waters Acquity Arc high-performance liquid chromatograph (HPLC). The HPLC conditions were based on a method developed by Wang et al. [19]. An LC-NH2 amino column (250 mm × 4.6 mm, Waters Corp., Milford, MA, USA) was used as the chromatographic column. The mobile phase consisted of acetonitrile and water in a ratio of 3:1, with a flow rate of 1.0 mL·min^-1^. The column temperature was maintained at 30 °C, and the injection volume was set to 20 µL.

The extraction of sucrose synthase synthesis direction (SuSy-s), sucrose synthase decomposition direction (SuSy-c), sucrose phosphate synthase (SPS), acid invertase (AI), and neutral invertase (NI) enzymes, as well as the determination of their activity, followed the method described by Joshi S [20]. A total of 3.5 mL of extract was added to 0.7 g of pulp powder and thoroughly ground. The extract consisted of a 50 mM Hepes-NaOH buffer (pH 7.5) containing 2.5 mM 1,4-Dithiothreitol (DTT), 5 mM MgCl2, 0.05% (v/v) TritonX-100, 1 mM Ethylenedinitrilotetraacetic acid (EDTA), 0.1% (w/v) Bovine Serum Albumin (BSA), and 2% (w/v) Polyvinyl pyrrolidone (PVP). After centrifugation at 13,000 g at 4 °C for 10–15 min, the supernatant was desalted using a Sephadex G25 PD-10 desalination column, and the desalted enzyme extract was stored at 4 °C for enzyme activity determination.

SPS activity determination: A 1.3 mL enzyme solution was mixed with 50 µL of the test reaction solution consisting of 50 mM Hepes-NaOH buffer (pH 7.5), 15 mM MgCl_2_, 1.0 mM Ethylenedinitrilotetraacetic acid (EDTA), 16 mM Uridine diphosphate glucose (UDPG), 4.0 mM Fructose-6-phosphate (F-6-P), and 20 mM Glucose-6-phosphate (G-6-P). The reaction was conducted at 32 °C for 30 min, followed by the addition of 50 µL of 5.0 M NaOH to stop the reaction. Subsequently, the enzyme was inactivated by boiling in a water bath for 10 min. After cooling, 1.5 mL of 36% HCl and 0.5 mL of 0.1% resorcinol were added. The reaction proceeded at 40 °C for 30 min, resulting in a light tea color. Following cooling, the absorbance was recorded at 480 nm using an ultraviolet-visible spectrophotometer. The enzyme activity was determined based on the sucrose standard curve (1.0 mg·mL^-1^). SuSy-s activity determination: 1.3 mL of the enzyme solution was mixed with 50 µL of the test reaction solution containing 80 mM Hepes-NaOH buffer (pH 8.5), 100 mM fructose, and 15 mM Uridine diphosphate glucose (UDPG). Following a 30 min reaction at 32 °C, 50 µL of 5.0 mM NaOH was added to halt the reaction. Subsequently, the reaction was conducted in a boiling water bath for 10 min. After cooling, 1.5 mL of 36% HCl and 0.5 mL of 0.1% resorcinol were added. The reaction was then carried out at 40 °C for 30 min, resulting in a pink color. Upon cooling, the absorbance at 480 nm was measured using an ultraviolet-visible spectrophotometer. The enzyme activity (µmol·g^-1^·min^-1^FW) was determined based on the standard curve of sucrose (1.0 mg·mL^-1^). SuSy-c activity determination: By combining 1.0 mL of enzyme solution with 1.0 mL of extract, followed by the addition of 490 µL of the test reaction solution containing 100 mM Phosphate buffered saline (PBS) (pH 6.0), 100 mM sucrose, and 5 mM Uridine diphosphate (UDP). The reaction was incubated at 30 °C for 1 h, and then 490 µL of 3,5-Dinitrosalicylic acid (DNS) reagent was added to stop the reaction, resulting in a yellow color. Subsequently, the reaction mixture was heated in a boiling water bath for 5 min, resulting in no color change for the blank and dark orange color for the other sample. After cooling, the absorbance was measured at 520 nm using an ultraviolet-visible spectrophotometer. The enzyme activity was calculated based on the standard curve of glucose (1.0 mg·mL^-1^). AI activity determination: By adding 1.0 mL of enzyme extract to 490 µL of a reaction solution consisting of 30 mM potassium acetate buffer (pH 5.0) and 200 mM sucrose. The reaction took place at 30 °C for 30 min, following which 490 µL 3,5-Dinitrosalicylic acid (DNS) reagent was added to stop the reaction. Subsequently, the absorbance at 540 nm was measured using an ultraviolet-visible spectrophotometer. The enzyme activity (µmol·g^-1^·min^-1^ FW) was calculated based on the standard curve of glucose (1.0 mg·mL^-1^). NI activity determination: By combining 2.0 mL of enzyme solution with 490 µL of a reaction solution containing 40 mM Hepes buffer (pH 7.5) and 200 mM sucrose, and incubating the mixture in a water bath at 30 °C for 30 min. Subsequently, 490 µL of 3,5-Dinitrosalicylic acid (DNS) was added to terminate the reaction, followed by centrifugation at 13,000 rpm for 8 min and heating in a boiling water bath for 5 min (except for the control, which exhibited different colors). The supernatant was collected, and the absorbance was measured at 540 nm using an ultraviolet-visible spectrophotometer. The enzyme activity (µmol·g^-1^·min^-1^FW) was calculated based on the standard curve of glucose (1.0 mg·mL^-1^).

### Determination of organic acid components and enzyme activities related to organic acid metabolism in fruits

The organic acid components were analyzed through high-performance liquid chromatography. The concentrations of oxalic acid, tartaric acid, shikimic acid, fumaric acid, citric acid, and malic acid in fruits were determined according to Ma et al. [21]. The grape pulp was rapidly ground into powder with liquid nitrogen. Subsequently, 1.5 g of the sample was weighed and mixed with 7.5 mL of ultrapure water. After centrifuging at 10,000 rpm for 10 min, the supernatant was filtered through a 0.22 μm aqueous microporous membrane, and the resulting filtrate was transferred to the sample bottle for testing. A column with specifications of Atlantis T3 (4.6 mm × 150 mm, 3 μm) was employed for the analysis. The mobile phase consisted of a 20 mmol·L^-1^ NaH_2_PO_4_ solution, with the pH adjusted to 2.7 using H_3_PO_4_. The flow rate was set at 0.50 mL·min^-1^, while the column temperature was maintained at 30℃. Detection was carried out at a wavelength of 210 nm, and the injection volume was 20 µL.

The extraction and activity determination of Citric acid synthase (CS), Cytoplasmic aconitase acid enzyme (Cyt-ACO), Mitochondrial aconitase acid enzyme (Mit-ACO), NAD-isocitrate dehydrogenase (NAD-IDH), Phosphoenolpyruvate carboxylase (PEPC), NAD-malate dehydrogenase (NAD-MDH), and NADP-malic enzyme (NADP-ME) enzymes followed the method described by Tang Mi [22]. All procedures were conducted at 4 ℃. Samples (3g) were weighed in a mortar, fruit pulp was extracted with 5 mL of grinding buffer [200 mM Tris-HCl (pH 8.2), 600 mM sucrose, 10 mM isoascorbic acid] and ground with a mortar and pestle. The mixture was centrifuged at 4000 g. The supernatant was then collected and re-centrifuged. Both supernatant and pellet were used to assay enzyme activity. The supernatant was diluted to 5 mL with an extracting buffer [200 mM Tris–HCl (pH 8.2), 10 mM isoascorbic acid, 0.1% Triton X-100]. Next, 2 mL of the diluted supernatant was centrifuged at 15,000 g for 15 min at 4 ℃. The resulting pellet was diluted to 2 mL with an extracting buffer for ACO and IDH assays. The remaining 3 mL of the diluted supernatant was further diluted to 6 mL with the extracting buffer, after which 2 mL of this was used for MDH and ME assays. The remaining 4 mL underwent dialysis with the extracting buffer for 10 h and then used for PEPC and CS assays. The specific determination system is as follows: ACO activity was determined in a 0.5 mL mixture composed of 40 mM Tris-HCl (pH 7.5), 100 mM NaCl, and 200 mM cis-aconitate. IDH activity was determined in a 0.5 mL mixture composed of 40 mM Hepes (pH 8.2), 800 mM Nicotinamide adenine dinucleotide (NAD), 200 mM MnSO_4_, and 2 mM isocitrate. MDH activity was determined in a 0.5 mL mixture composed of 40 mM Tris-HCl (pH 8.2), 2 mM MgCl_2_, 10 mM KHCO_3_, 500 mM Glutathione (GSH), 150 mM Nicotinamide adenine dinucleotide (NADH), and 2 mM oxaloacetate (OAA). ME activity was determined in a 0.5 mL mixture composed of 80 mM Tris-HCl (pH 7.4), 170 mM Nicotinamide adenine dinucleotide phosphate (NADP), 200 mM MnSO_4_, and 2 mM malate. PEPC activity was determined in a 0.5 mL mixture composed of 40 mM Tris-HCl (pH 8.5), 2 mM MgCl_2_, 10 mM KHCO_3_, 500 mM Glutathione (GSH), 150 mM Nicotinamide adenine dinucleotide (NADH), and 2 mM Phosphoenolpyruvate (PEP). CS activity was assayed in a 0.5 mL mixture composed of 40 mM Tris-HCl (pH 9.0), 40 mM 5,5’-Dithiobis-(2-nitrobenzoic acid) (DTNB), 40 mM acetyl-CoA, and 4 mM Oxaloacetic acid (OAA). All the reactions were started by the addition of the respective enzyme extracts. The changes per minute in absorption were recorded by a spectrophotometer (Shimadzu UV-2401, Japan).

### Determination of the aroma components of the fruit

At the maturity stage, 105 d after the initial flowering, a total of 30 grapes were selected from the fruit samples of the same maturity. The stems and seeds were removed, and the pulp was homogenized using a homogenizer. The SPME-GC-MS procedure followed the methodology outlined in previous studies [23]. For each sample, 9 g of pulp was mixed with 2-Octanol (35 µL, 8.82 mg·L^-1^) as an internal standard and NaCl (1.5 g) in a 20 mL glass vial, which was then capped. The sample was equilibrated at 50 °C for 10 min before extraction with an SPME fiber for 30 min. After extraction, the fiber was immediately desorbed into the GC injection port at 260 °C for 5 min in splitless mode. The aroma compounds were separated and identified using an Agilent 7890 GC (Agilent Technologies, Santa Clara, CA) coupled with an Agilent 5975 C MS.

The content of each aroma substance(µg·kg^-1^)=(A1/A2)·(M1/M2)·1 000, A1 was the peak area of the substance to be measured; A2 was the peak area of internal standard; M1 was the mass of internal standard, µg; M2 was sample mass, g.

### Statistical analysis of data

All parameters were measured in a minimum of three replications and are presented as means ± standard deviation (Data show the mean ± SE (*n* = 3).SPSS 23.0 was used for data processing, and Duncan ‘s multiple comparison was used for analysis of variance. Excel 2016 and Origin 2022 were used for chart drawing.

## Results

### Effects of pro-ca on endogenous hormone content in ‘chardonnay’ fruit

**Fig. 1:**
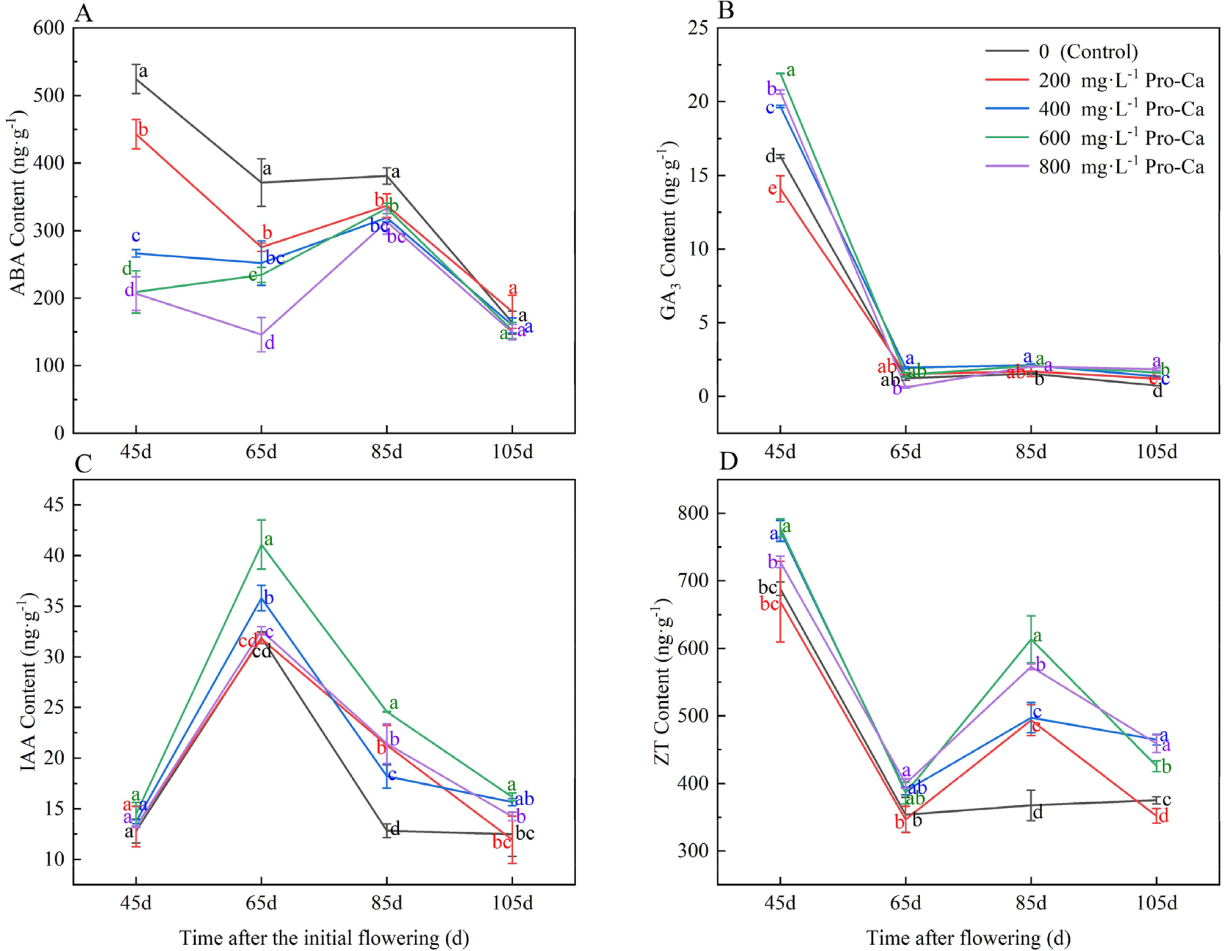
Effect of Pro-Ca on endogenous hormone content in ‘Chardonnay’ grape fruit. **(A)** ABA: abscisic acid. **(B)** GA_3_: gibberellic acid. **(C)** IAA: auxin. **(D)** ZT: zeatin. Results are means ± SE of three independent replications. Different letters represent significant differences between treatments (*p* < 0.05)

During the development process of grapes from young fruit to maturity, the ABA content in the fruit showed a pattern of first decreasing, then increasing, and finally decreasing again (Fig. 1A). Except during the ripening stage, different concentrations of Pro-Ca treatment reduced ABA content and promoted fruit ripening at different growth stages compared to the control. Among these treatments, 600 mg·L^-1^ Pro-Ca treatment and 800 mg·L^-1^ Pro-Ca treatment had a significant effect on reducing ABA content in grape berries.

GA_3_ content in grape fruit decreased as the fruit developed from setting to ripeness, with a sharp decline observed 65 days after first flowering. GA_3_ content tended to stabilize in the later stages of fruit growth and development (Fig. 1B). Pro-Ca treatment significantly increased GA_3_ content at 45 d(days)after the initial flowering. It is noteworthy that treatment with 600 mg·L^-1^ Pro-Ca showed the most significant improvement effect with an increase of 34.62% compared to the control.

The IAA content in grape fruit initially increased and then decreased as the fruit ripened (Fig. 1C). Pro-Ca treatment significantly increased IAA content at 65 d, 85 d and 105 d after the initial flowering. The 600 mg·L^-1^ Pro-Ca treatment showed higher IAA content than other treatments at these time points, showing an increase of 28.79%, 91.71% and 29.51% compared to the control, respectively.

As grape fruit ripening progressed, the ZT content in the control group first decreased and then increased, while the Pro-Ca treatment group showed a pattern that first decreased, then increased, and finally decreased (Fig. 1D). In particular, the Pro-Ca treatments of 400 mg·L^-1^ and 600 mg·L^-1^ significantly increased the ZT content 45 d after the initial flowering, resulting in an increase of 12.45% and 13.08% in comparison led to control. At 65 d after the initial flowering, the grape fruits treated with 800 mg·L^-1^ Pro-Ca had the highest ZT content and showed an increase of 13.04% compared to the control. At 85 d after the initial flowering, the Pro-Ca treatments of 600 mg·L^-1^ and 800 mg·L^-1^ increased the ZT content by 66.98% and 55.84%, respectively, compared to the control.

### Effects of pro-ca on total soluble sugar and sugar components of ‘chardonnay’ fruits

**Fig. 2:**
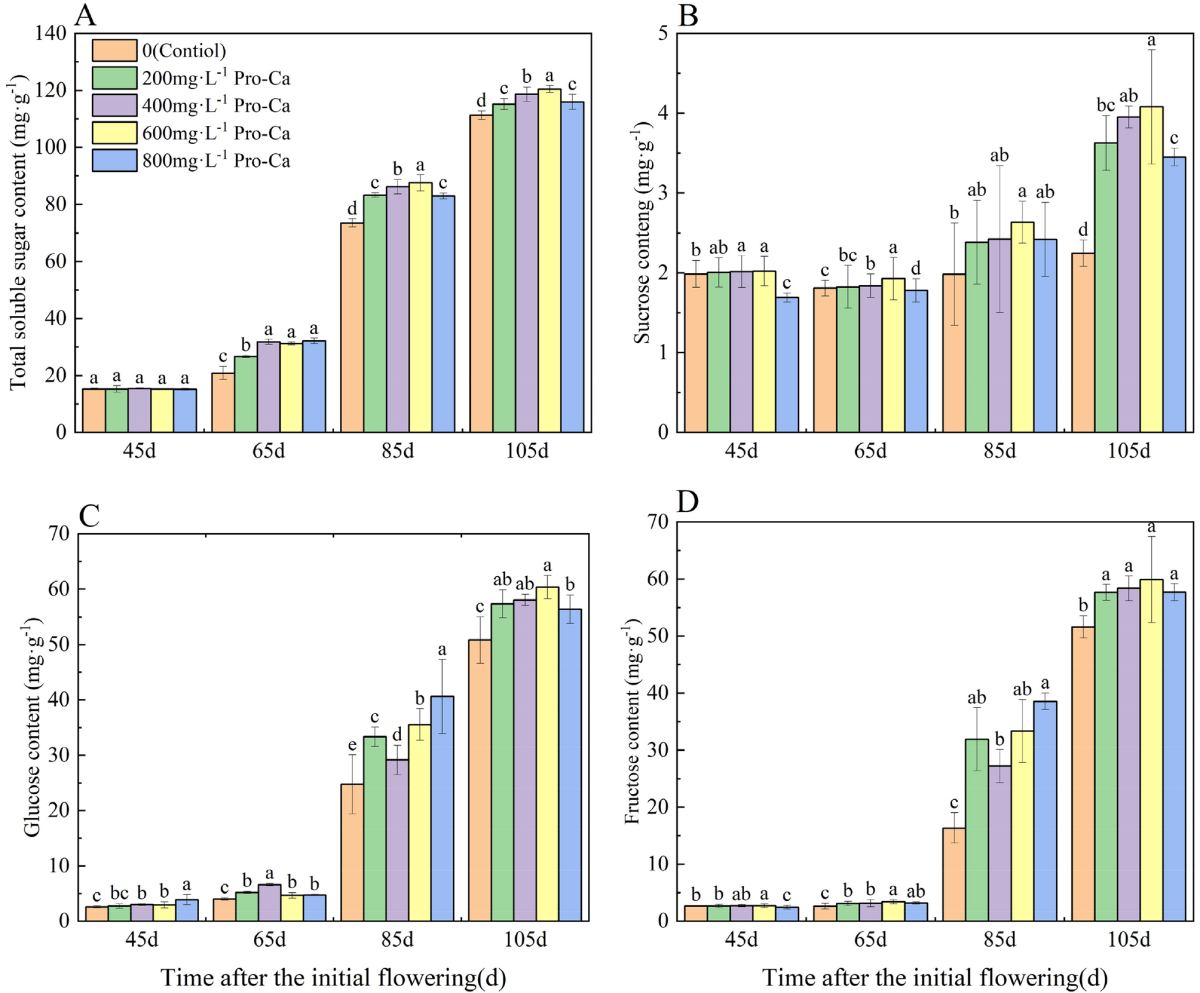
Effects of Pro-Ca on total soluble sugar and sugar components of ‘Chardonnay’ fruit. **(A)** Total soluble sugar. **(B)** Sucrose. **(C)** Glucose. **(D)** Fructose. Results are means ± SE of three independent replications. Different letters represent significant differences between treatments (*p* < 0.05)

Total soluble sugar content in grape fruit gradually increased as the fruit matured (Fig. 2A), with a rapid increase observed 85 d after initial flowering. Compared to the control, Pro-Ca treatment increased total soluble sugar content of fruits at 65 d, 85 d and 105 d after initial flowering. Among these treatments, the total soluble sugar content of grapefruit treated with 600 mg·L^-1^ Pro-Ca was highest at 85 d and 105 d after the initial flowering, showing an increase of 19.18% and 11.28%, respectively, compared to the Control.

Grape sucrose content also increased gradually as the fruit matured (Fig. 2B), with the greatest increase observed 105 d after the initial flowering. Among the different treatments, 600 mg·L^-1^ Pro-Ca treatment had the most significant effect at 45 d, 65 d, 85 d and 105 d after the initial flowering, resulting in increases of 1.56%, 6.44%, 33.03% and 81.28% compared to the control.

During the period from the initiation of young fruit to full maturity, there was an observable increase in the levels of glucose and fructose within grape berries (Fig. 2C, D). This increase occurred particularly rapidly 85 d after the first flowering. The application of Pro-Ca demonstrated a significant effect on enhancing glucose and fructose levels in grape berries at 85 and 105 d after initial flowering. Specifically, grape berries treated with 800 mg·L^-1^ Pro-Ca exhibited higher contents of glucose and fructose at 85 d after initial flowering compared to other treatments. Additionally, grape berries treated with 600 mg·L^-1^ Pro-Ca at 105 d after initial flowering also showed higher contents of glucose and fructose compared to other treatments.

### Effects of pro-ca on the activity of sugar metabolism enzymes in ‘chardonnay’ fruit

**Fig. 3:**
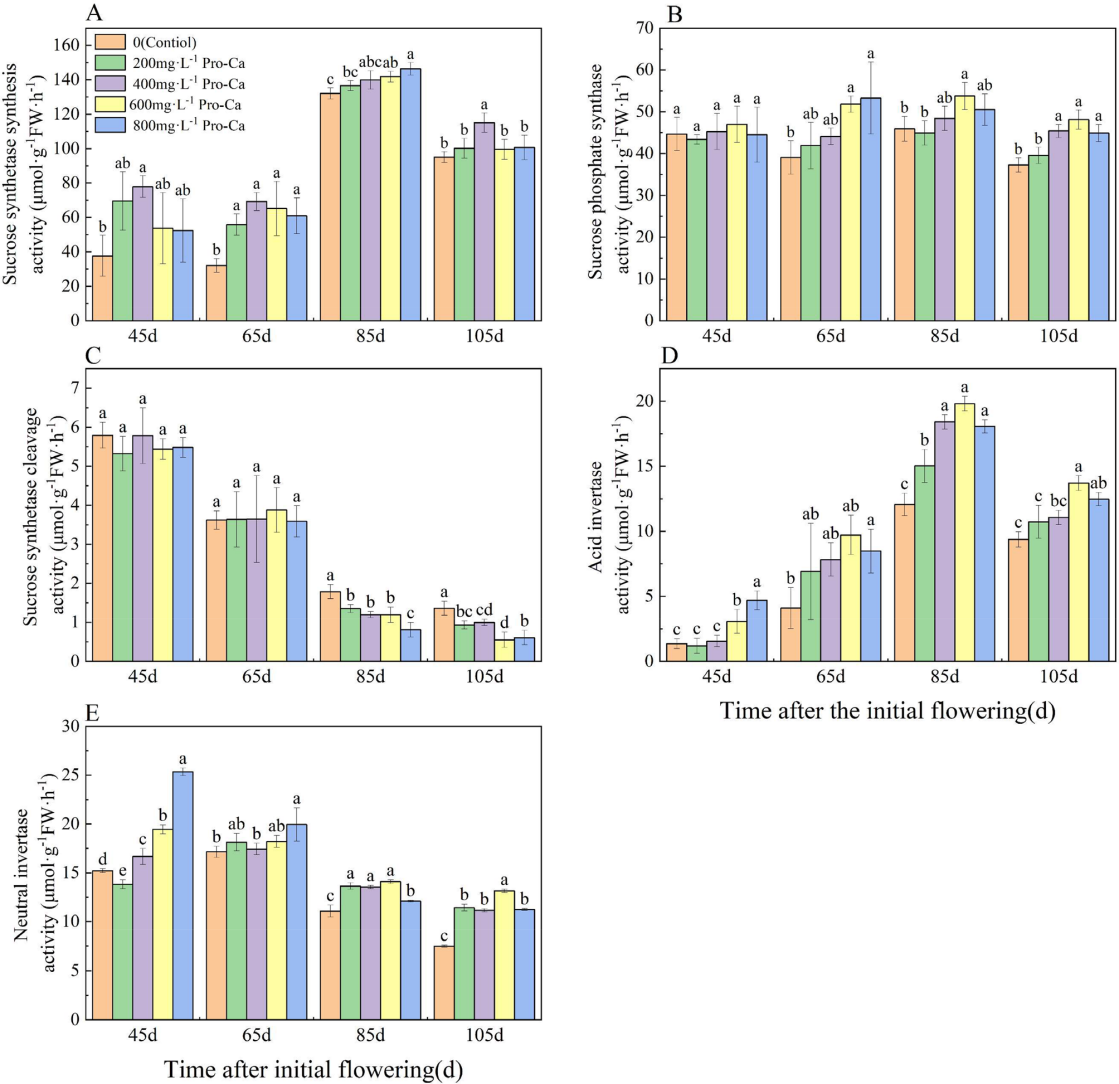
Effect of Pro-Ca treatment on the activity of sugar metabolism enzymes in ‘Chardonnay’ fruit. **(A)** SuSy-s: sucrose synthase synthesis. **(B)** SuSy-c: sucrose synthase cleavage. **(C)** SPS: sucrose phosphate synthase. **(D)** AI: acid invertase. **(E)** NI: neutral invertase. Results are means ± SE of three independent replications. Different letters represent significant differences between treatments (*p* < 0.05)

The activity of SuSy-s in each treatment decreased gradually from young fruit to mature fruit (Fig. 3A). Different concentrations of Pro-Ca treatment increased the activity of SuSy-s in the fruit. Among them, the activity of SuSy-s in the fruit treated with 600 mg·L^-1^ Pro-Ca was significantly higher than the other treatments at 105 d after the initial flowering, showing a 21.08% increase compared to the control group. The SPS activity of grape fruit did not show significant changes from young fruit to maturity (Fig. 3B). However, when comparing with the control group, the SPS activity of grape fruit treated with 600 mg·L^-1^ Pro-Ca at 85 and 105 d after the initial flowering was higher than the other treatments by 24.23% and 45.99%, respectively.

As grape berries matured, the SuSy-c activity exhibited a gradual decrease across all treatments (Fig. 3C). During the final stages of fruit development, just before harvest, the application of Pro-Ca had a diminishing effect on SuSy-c activity. At 45 and 65 d after the initial flowering, no significant differences were observed between the treatment and control groups. However, by 85 d after the initial flowering, there was a reduction in SuSy-c activity with an increase in Pro-Ca concentration, and the SuSy-c activity in the various Pro-Ca treatment groups was notably lower than that of the control group. Specifically, when compared to the control, there were reductions in SuSy-c activity by 24.40%, 33.01%, 33.17%, 54.55%, and 27.34%, respectively. By 105 d after the initial flowering, the SuSy-c activity of the fruits treated with 600 mg·L^-1^ Pro-Ca was the lowest, exhibiting a 59.36% decrease compared to the control group.

Grapefruit AI activity gradually increased as the fruit matured (Fig. 3D), and AI activity was significantly higher in ripe fruits compared to young fruits. Notably, fruits treated with 600 mg·L^-1^ Pro-Ca at 65, 85, and 105 d after initial flowering showed significantly higher AI activity compared to other treatments, with increases of 137.30%, 64 0.22% and 46.23% respectively. During the gradual maturation of grape fruits, except for the decrease in NI activity observed in fruits treated with 600 and 800 mg·L^-1^ Pro-Ca, the NI activity of fruits treated with other methods showed an initial increase followed by a decrease (Fig. 3E). Among them, the NI activity of the fruits treated with 800 mg·L-1 Pro-Ca was significantly higher than the other treatments at 45 d after the initial flowering, with a 66.59% increase compared to the control. At 85 and 105 d after the initial flowering, the NI activity of the fruit treated with 600 mg·L^-1^ Pro-Ca was the highest and significantly higher than the control group, with increases of 27.34% and 75.51%, respectively.

### Effects of pro-ca on the content of organic acid components in ‘chardonnay’ fruit

**Fig. 4:**
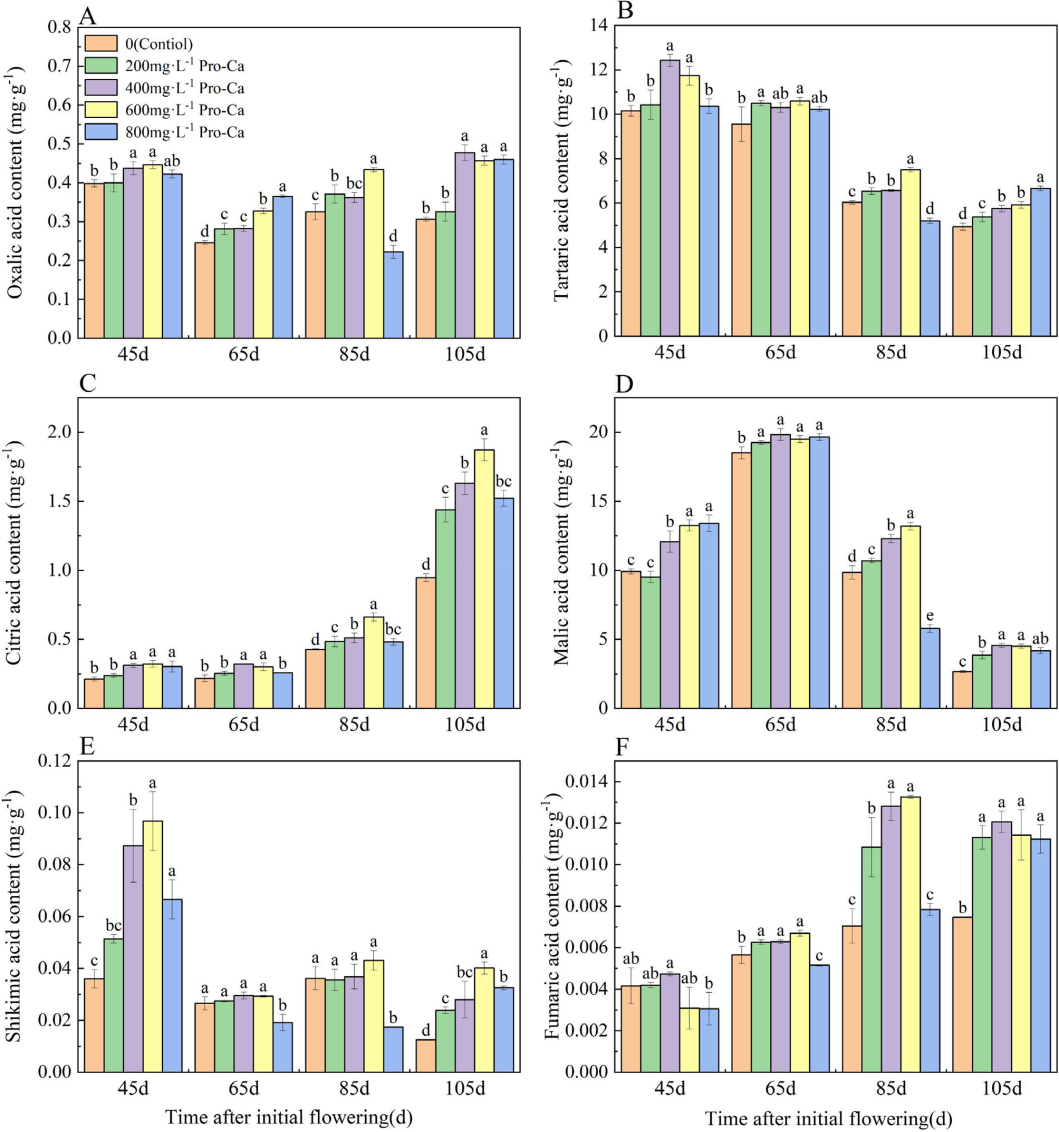
Effect of Pro-Ca on the content of organic acid components in ‘Chardonnay’ fruit. **(A)** Oxalic acid. **(B)** Tartaric acid. **(C)** Citric acid. **(D)** Malic acid. **(E)** Shikimic acid. **(F)** Fumaric acid. Results are means ± SE of three independent replications. Different letters represent significant differences between treatments (*p* < 0.05)

Grape fruit oxalic acid content showed an initial decrease in each treatment and then an increase as the fruits ripened (Fig. 4A). Pro-Ca treatment increased oxalic acid content compared to control. In particular, 105 d after the initial flowering, the oxalic acid content increased significantly in the fruits treated with 400 mg·L^-1^ Pro-Ca, 600 mg·L^-1^ Pro-Ca and 800 mg·L^-1^ Pro-Ca by 56.02%, 49.38% and 50.23% compared to the control.

The tartaric acid content in the fruit decreased gradually from young fruit to mature fruit in all treatments (Fig. 4B). Pro-Ca treatment increased the tartaric acid content in the fruit compared to the control. Specifically, at 65 and 85 d after the initial flowering, the treatment with 600 mg·L^-1^ Pro-Ca resulted in the highest tartaric acid content, which was significantly higher than that of the control (*P* < 0.05).

The citric acid content of grape fruit increased gradually as the fruit matured (Fig. 4C), with a rapid increase at 105 d after the initial flowering (maturity stage). Pro-Ca treatment increased the citric acid content at each stage. Notably, at 105 d after the initialflowering, the fruit treated with 600 mg·L^-1^ Pro-Ca had significantly higher citric acid content compared to other treatments, showing a 97.80% increase compared to the control.

The malic acid content of grape fruits in each treatment (Fig. 4D) initially increased and then decreased as the fruits grew and developed, reaching its peak at 65 d after the initial flowering, and sharply decreasing at 105 d after the initial flowering. At 105 d after the initial flowering, the malic acid content of grape fruits treated with different concentrations of Pro-Ca was significantly higher than that of the control. Specifically, it was 44.29%, 70.51%, 68.87%, and 56.56% higher than the control for different Pro-Ca concentrations, respectively.

The contents of shikimic acid and fumaric acid in the fruits were low (Fig. 4E, F), and the fumaric acid content gradually increased as the shikimic acid content decreased in mature fruits (Fig. 4E). At 85 d after the initial flowering, the contents of shikimic acid and fumaric acid in fruits treated with 600 mg·L^-1^ Pro-Ca were higher than those in other treatments, being 119.02% and 118.21% of that in the control, respectively.

### Effects of pro-ca on the activities of enzymes related to organic acid metabolism in ‘chardonnay’ fruit

**Fig. 5:**
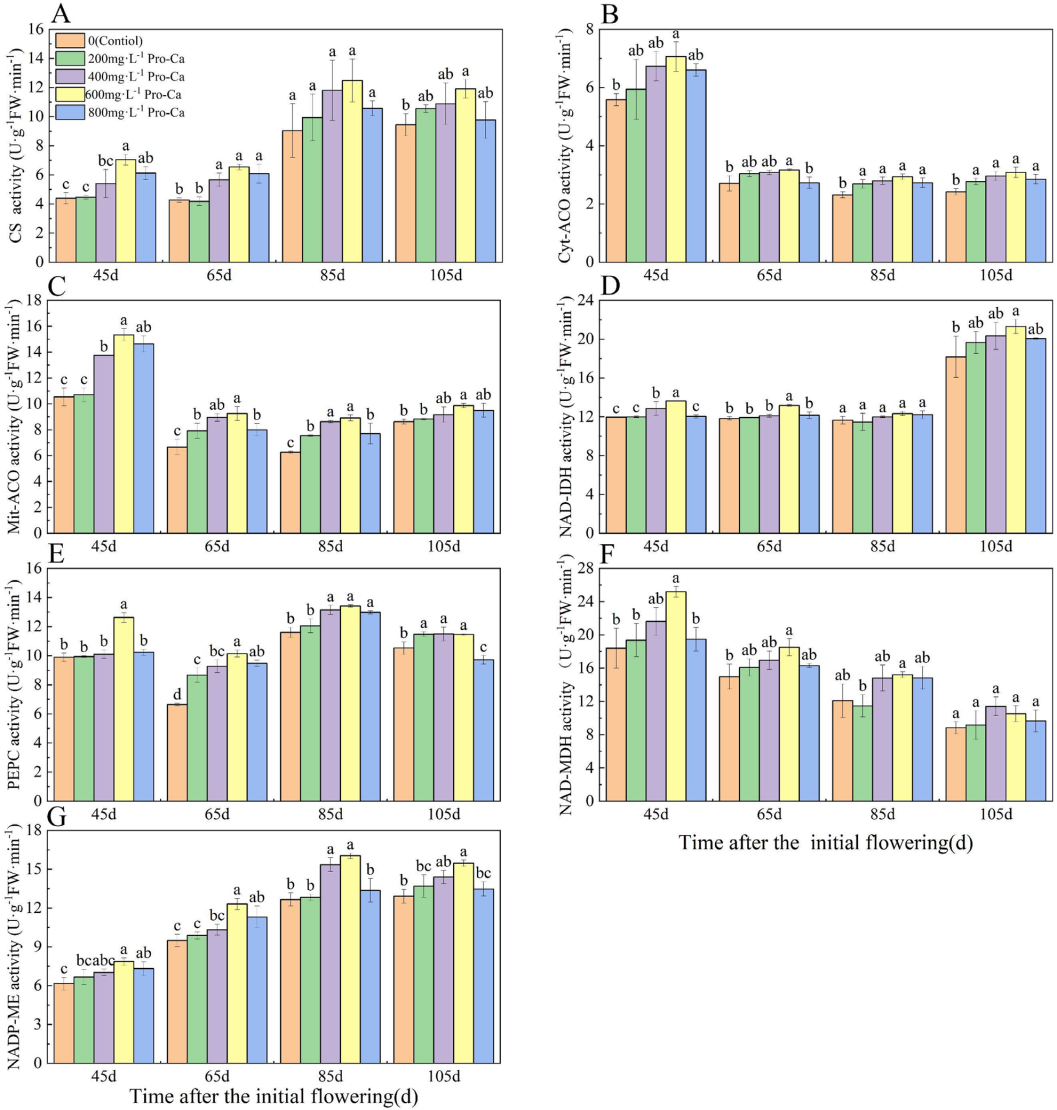
Effects of Pro-Ca on the activities of enzymes related to organic acid metabolism in ‘Chardonnay’ fruit. **(A)** CS: citrate acid synthase. **(B)** Cyt-ACO: cytoplasmic aconitic acid enzyme. **(C)** Mit-ACO: mitochondrial aconitic acid enzyme. **(D)** NAD-IDH: NAD-isocitrate dehydrogenase. **(E)** PEPC: phosphoenolpyruvate carboxylase. **(F)** NAD-MDH: NAD-malate dehydrogenase **(F)** NADP-ME: NADP-malate enzyme. Results are means ± SE of three independent replications. Different letters represent significant differences between treatments (*p* < 0.05)

During the process of the grape from young fruit to maturity, CS activity showed a trend of slow decrease, fast increase, and slow decrease in each treatment (Fig. 5A). Activity was highest at 85 d after the initial flowering and slowly decreased at 105 d after the initial flowering (maturity stage). Different concentrations of Pro-Ca treatment in each period increased the CS activity of the fruit. Among them, the CS activity of 600 mg·L^-1^ Pro-Ca treatment was highest at 45 d, 65 d, 85 d and 105 d after the initial flowering and was 60.19%, 53.17%, 38, 02% and 26.21% higher than that of the control. ACO catalyzes citric acid to produce water and cis-aconitic acid. Cyt-ACO and Mit-ACO are two isozymes in plants. The activities of Cyt-ACO and Mit-ACO were highest 45 d after the initial flowering, decreased rapidly, and then increased slowly with fruit development (Fig. 5B, C). Compared to control, Pro-Ca treatment increased the activities of Cyt-ACO and Mit-ACO. The activities of Cyt-ACO and Mit-ACO were highest in the treatment with 600 mg·L^-1^ Pro-Ca at 45 d, 65 d, 85 d and 105 d after the initial flowering. NAD-IDH is also a control factor for citric acid degradation. NAD-IDH activity (Fig. 5D) showed a trend that initially decreased slowly and then increased rapidly during fruit development. NAD-IDH activity was highest 105 d after initial flowering (maturity). At 45 d and 65 d after the initial flowering, the NAD-IDH activity of the 600 mg·L^-1^ Pro-Ca treatment was significantly higher (*P* < 0.05) than that of other treatments, which increased by 14.10% and was 11.54% higher than that of the control.

With the development and maturity of the fruit, the PEPC activity of the fruit decreased first, then increased and then decreased (Fig. 5E). With the increase of Pro-Ca treatment concentration, the PEPC activity of the fruit increased first and then decreased at each stage. Among them, the PEPC activity of 600 mg·L^-1^ Pro-Ca treatment was the highest at each stage, and the PEPC activity of 600 mg·L^-1^ Pro-Ca treatment was significantly higher than that of other treatments at 45 d after the initial flowering (*P* < 0.05), which was 27.58% higher than that of the control. The NAD-MDH activity decreased gradually during fruit development (Fig. 5F). Pro-Ca treatment increased the NAD-MDH activity of fruit. Among them, the NAD-MDH activity of 600 mg·L^-1^ Pro-Ca treatment was the highest at 45 d, 65 d and 85 d after the initial flowering, which was 36.82%, 23.54% and 25.85% higher than that of the control, respectively. The activity of NADP-ME increased first and then decreased during fruit development (Fig. 5G). The activity of NADP-ME decreased at 105 d after the initial flowering (maturity stage). With the increase of Pro-Ca treatment concentration in each period, the activity of NADP-ME in fruits increased first and then decreased. Among them, the activity of NADP-ME treated with 600 mg·L^-1^ Pro-Ca was the highest in each period.

### The impact of pro-ca on the volatile aroma compounds of ‘chardonnay’ grape berries

Under the condition of HS-SPME-GC-MS, we analyzed the aroma substances of ‘Chardonnay’ grape fruits treated with different concentrations of Pro-Ca at 105 d after initial flowering (maturity stage). The results are presented in Table 1.

Table 1 shows that a total of 36 aroma substances were isolated and identified. Among these, 18 aroma substances were present in all treatments. The levels of aldehydes, esters, alcohols, phenols, acids, ketones, and terpenes were significantly higher in the 600 mg·L^-1^ Pro-Ca treatment compared to the control. Specifically, compared with the control group, the levels of aldehydes increased by 56.72%, esters by 423.85%, alcohols by 89.51%, phenols by 266.54%, acids by 211.62%, ketones by 51.46%, and terpenes by 83.96%. In the control group, 11 types of aldehydes were detected. However, Pro-Ca treatment reduced the number of aldehyde types and increased the types of esters, alcohols, and phenols. Each treatment only had one type of acids and terpenes, while ethers and alkenes were only found in the control group.

**Table 1:** Effect of exogenous Pro-Ca on aroma volatiles of ‘Chardonnay’ grape berries

Varieties	No.	Molecular formula	CAS	Compound	Content of each aroma component/(µg·kg-1)				
					0(Control)	200 mg·L^-1^ Pro-Ca	400 mg·L^-1^Pro-Ca	600 mg·L^-1^Pro-Ca	800 mg·L^-1^Pro-Ca
Aldehydes	1	C9H18O	124-19-6	Nonanal	29.28 ± 0.91c	31.31 ± 3.83bc	33.26 ± 6.33b	42.53 ± 2.7a	22.3 ± 0.8d
	2	C12H24O	112-54-9	Dodecanal	179.25 ± 11.22a	73.12 ± 4.79b	65.29 ± 5.6b	32.81 ± 0.97c	22.32 ± 1.65d
	3	C8H8O	122-78-1	Benzeneacetaldehyde	313.95 ± 8.02e	827.73 ± 58.55d	1298.5 ± 183.84c	2129.05 ± 113.17a	2075.41 ± 132.38b
	4	C10H20O	112-31-2	Decanal	74.43 ± 0.96c	188.02 ± 45.28a	103.02 ± 8.18b	33.92 ± 1.63d	30.79 ± 0.35d
	5	C6H12O	15877-57-3	Pentanal, 3-methyl-	510.71 ± 13.66a	455.52 ± 20.6b	355.32 ± 24.59c	178.06 ± 14.31d	105.96 ± 0.64e
	6	C15H22O2	1620-98-0	3,5-di-tert-Butyl-4-hydroxybenzaldehyde	18.48 ± 1.11d	89.42 ± 3.46a	45.5 ± 3.12b	33.91 ± 2.14c	28.98 ± 0.55c
	7	C3H4O	107-02-8	2-Propenal	1102.03 ± 96.58a	/	/	/	/
	8	C10H16O	25152-84-5	2,4-Decadienal, (E,E)-	169.75 ± 60.24b	262.9 ± 17.03a	/	/	/
	9	C8H6O2	623-27-8	1,4-Benzenedicarboxaldehyde	203.02 ± 0.5a	132.56 ± 12.36b	126.26 ± 56.57bc	104.63 ± 26.36c	96.24 ± 17.24c
	10	C6H6O3	67-47-0	5-Hydroxymethylfurfural	/	223.22 ± 34.55c	1124.27 ± 106.67a	1215.22 ± 102.35a	941.26 ± 74.91b
	11	C10H16O	25152-84-5	2,4-Decadienal, (E,E)-	16.97 ± 6.02a	5.61 ± 0.44b	/	/	/
	12	C6H12O	66-25-1	Hexanal	475.08 ± 27.79c	781.13 ± 61.61b	1075.1 ± 234.2a	1077.22 ± 203.22a	/
	Kind				11	11	9	9	8
	Sum				3092.95	3070.54	4226.52	4847.35	3323.26
Esters	13	C16H22O4	84-74-2	Dibutyl phthalate	54.36 ± 1.11c	197.59 ± 14.55a	59.66 ± 2.17b	51.01 ± 2.94 cd	50.9 ± 7.96d
	14	C15H14O3	890-98-2	Benzyl mandelate	44.92 ± 0.53a	37 ± 0.6b	30.75 ± 2.99c	/	/
	15	C12H14O4	84-66-2	Diethyl Phthalate	/	43.25 ± 0.85a	35.67 ± 2.89b	10.27 ± 1.2c	9 ± 0.38c
	16	C10H10O4	131-11-3	Dimethyl phthalate	/	/	109.48 ± 1.78c	304.07 ± 5.53a	191.41 ± 2.2b
	17	C8H8O3	119-36-8	Methyl salicylate	/	17.08 ± 5.83c	26.86 ± 5.65b	32.26 ± 9.21a	16.07 ± 0.78c
	18	C3H4O2	692-45-5	Formic acid, ethenyl ester	32.37 ± 0.95e	287.12 ± 29.7d	311.58 ± 164.4c	395.13 ± 33.23a	344.53 ± 26.95b
	19	C10H16O2	65405-80-3	2-Butenoic acid, 3-hexenyl ester, (E,Z)-	19.68 ± 2.62b	53.89 ± 2.1a	54.01 ± 2.5a	/	/
	Kind				4	6	7	5	5
	Sum				151.33	635.93	628.01	792.74	611.91
Alkanes	20	C7H14	108-87-2	Cyclohexane, methyl-	33.51 ± 2.93a	20.32 ± 5.61d	31.43 ± 2.53ab	34.38 ± 5.63a	24.41 ± 1.47c
	21	C10H22	17302-01-1	3-Ethyl-3-methylheptane	13.47 ± 0.77d	23.7 ± 3.65c	65.29 ± 2.3a	40.75 ± 1.63b	22.32 ± 1.65c
	Kind				2	2	2	2	2
	Sum				46.98	44.02	96.72	75.13	46.73
Alcohols	22	C10H18O	78-70-6	Linalool	8.63 ± 0.64e	18.06 ± 4.59c	28 ± 0.27b	38.56 ± 2.86a	16.1 ± 1.35d
	23	C10H18O	106-24-1	Geraniol	534.09 ± 3.22b	756.14 ± 90.4a	743.09 ± 15.58a	525.76 ± 12.81b	270.44 ± 3.57c
	24	C6H14O	111-27-3	1-Hexanol	/	52.96 ± 5.54c	148.08 ± 0b	330.42 ± 8.14a	667.57 ± 184.81a
	25	C8H10O	60-12-8	Phenylethyl Alcohol	95.79 ± 2.88d	167.32 ± 21.54c	260.07 ± 4.99b	315.27 ± 31.15a	101.41 ± 9.94d
	Kind				3	4	4	4	4
	Sum				638.51	994.48	1179.24	1210.01	1055.52
Phenols	26	C15H24O	128-37-0	Butylated Hydroxytoluene	52.6 ± 6.88d	64.56 ± 3.19c	92.96 ± 1.57b	104.85 ± 4.92a	40.61 ± 2.62e
	27	C9H12O2	2785-89-9	Phenol, 4-ethyl-2-methoxy-	/	15.22 ± 1.35c	25.63 ± 2.26b	39.52 ± 3.87a	13.44 ± 1.04d
	28	C10H12O2	97-53-0	Eugenol	/	14.8 ± 1.66c	23.18 ± 0.4b	48.43 ± 10.07a	6.16 ± 1.06d
	Kind				1	3	3	3	3
	Sum				52.6	94.58	141.77	192.8	60.21
Ethers	29	C7H14O	3739-64-8	Butane, 1-(2-propenyloxy)-	25.01 ± 4.49a	/	/	/	/
Alkenes	30	C8H16	5026-76-6	1-Heptene, 6-methyl-	1146.79 ± 82.92a	/	/	/	/
Acids	31	C12H14O4	2359-09-3	1,3-Benzenedicarboxylic acid, 5-(1,1-dimethylethyl)-	53.86 ± 10.68d	58.71 ± 11.18d	137.52 ± 19.91b	167.65 ± 45.9a	107.47 ± 16.28c
Ketones	32	C13H20O	79-77-6	trans-.beta.-Ionone	1707.85 ± 14.64d	2084.19 ± 491.73c	2174.91 ± 265.73b	2586.66 ± 202.49a	1977.64 ± 122.36c
Heterocyclic	33	C6H4O3	823-82-5	2,5-Furandicarboxaldehyde	/	32.29 ± 0.8c	66.47 ± 4.41a	67.83 ± 1.03a	40.89 ± 1.9b
	34	C10H16O	3777-70-6	Furan, 2-hexyl-	/	25.31 ± 2.4a	15.9 ± 3.12b	4.23 ± 0.24c	1.38 ± 0.09d
	35	C14H20O2	719-22-2	2,5-Cyclohexadiene-1,4-dione, 2,6-bis(1,1-dimethylethyl)-	/	181.63 ± 18.78a	125.22 ± 9.8b	114.54 ± 2.04c	102.18 ± 10.89c
	Kind				/	3	3	3	3
	Sum				/	239.23	207.59	186.6	144.45
Terpenes	36	C10H20O	1490-04-6	Cyclohexanol, 5-methyl-2-(1-methylethyl)-	123.84 ± 1.11e	163.32 ± 15.65c	199.47 ± 14.89b	227.81 ± 1.5a	137.75 ± 21.45d
	Kind				25	31	31	29	28
	Sum				7039.66	7385	8991.75	10286.75	7464.94

Notes: Results are means ± SE of three independent replications. Different letters represent significant differences between treatments (*p* < 0.05)

**Fig. 6:**
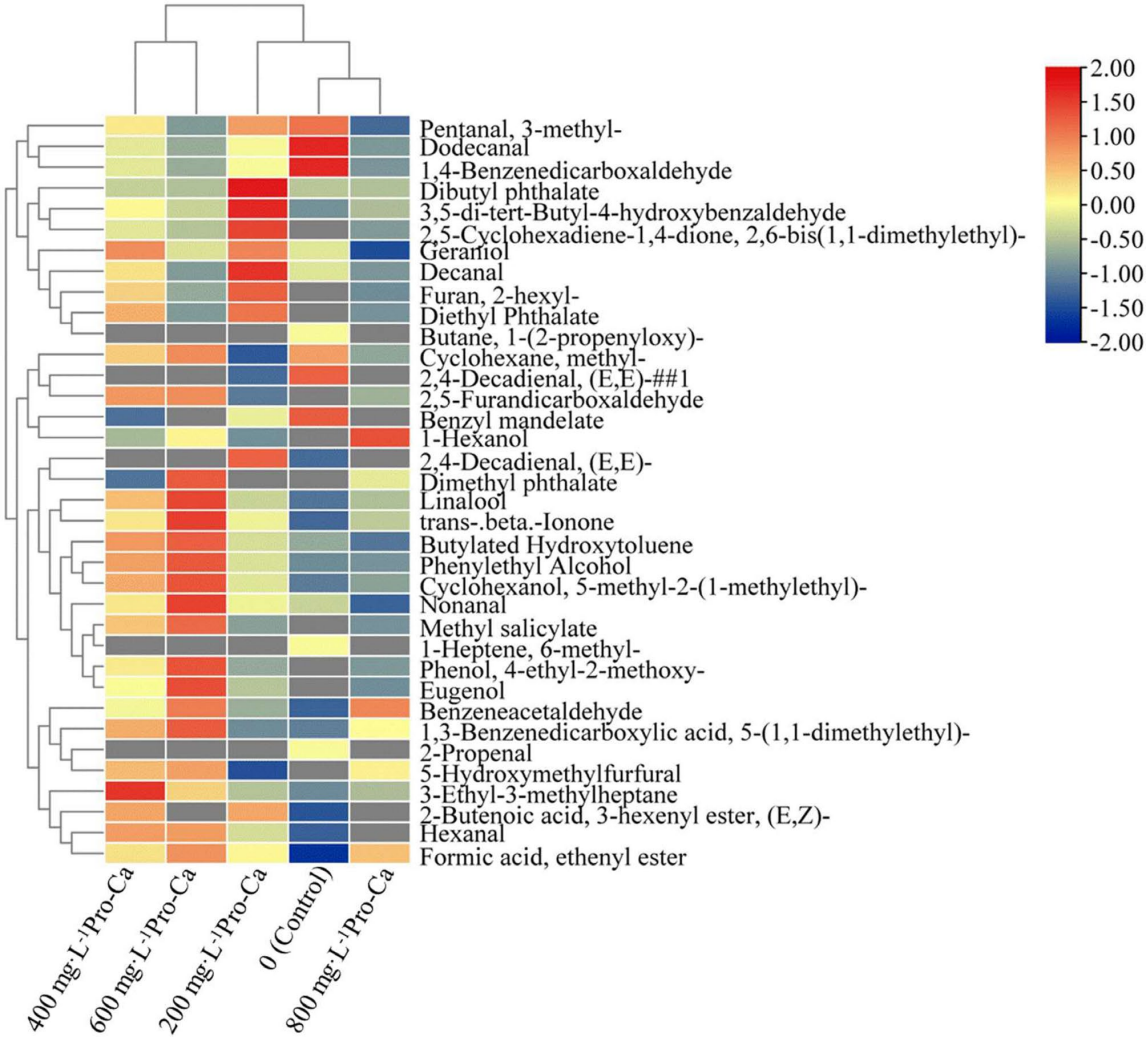
Cluster Heat Map of Volatile Aroma Components of ‘Chardonnay’ Fruit Treated with Exogenous Pro-Ca. The data used in the figure are the normalized original values of aroma volatiles. Blue indicates a lower content and red indicates a higher content

In order to further analyze the effect of exogenous Pro-Ca treatment on the volatile aroma substances of wine grape fruits, the original values of volatile aroma substances of the samples were analyzed by cluster heat map. The average content of aroma substances in each treatment was used to draw a cluster heat map. As shown in Fig. 6, it can be intuitively seen that there are significant differences in volatile aroma substances between the samples. Blue indicates low content, red indicates high content, and the darker the color, the higher the content of this component.

From Fig. 6, each treatment can be clustered into two categories, 600 mg·L^-1^Pro-Ca treatment and 400 mg·L^-1^Pro-Ca as a class, the control and other treatments as a class, indicating that 600 mg·L^-1^Pro-Ca treatment and 400 mg·L^-1^Pro-Ca have similar effects on volatile aroma substances in grape fruits, and there are significant differences with the control. In the cluster analysis of volatile aroma substances in wine grape berries, it was found that (Figs. 6), 2,4-Decadienal, (E, E) -, Dimethyl phthalate, Linalool, trans-.beta.-Ionone, Butylated Hydroxytoluene, Phenylethyl Alcohol, Cyclohexanol. 5-methyl-2- (1-methylethyl) -, Nonanal, Methyl salicylate, 1-Heptene, 6-methyl-, Phenol, 4-ethyl-2-methoxy-, Eugenol were clustered into one group, indicating that they had high similarity, and 600 mg·L^-1^ Pro-Ca treatment significantly increased the content of such substances.

### Correlation analysis between endogenous hormones, sugar and acid components and aroma substances in grape fruits

**Fig. 7:**
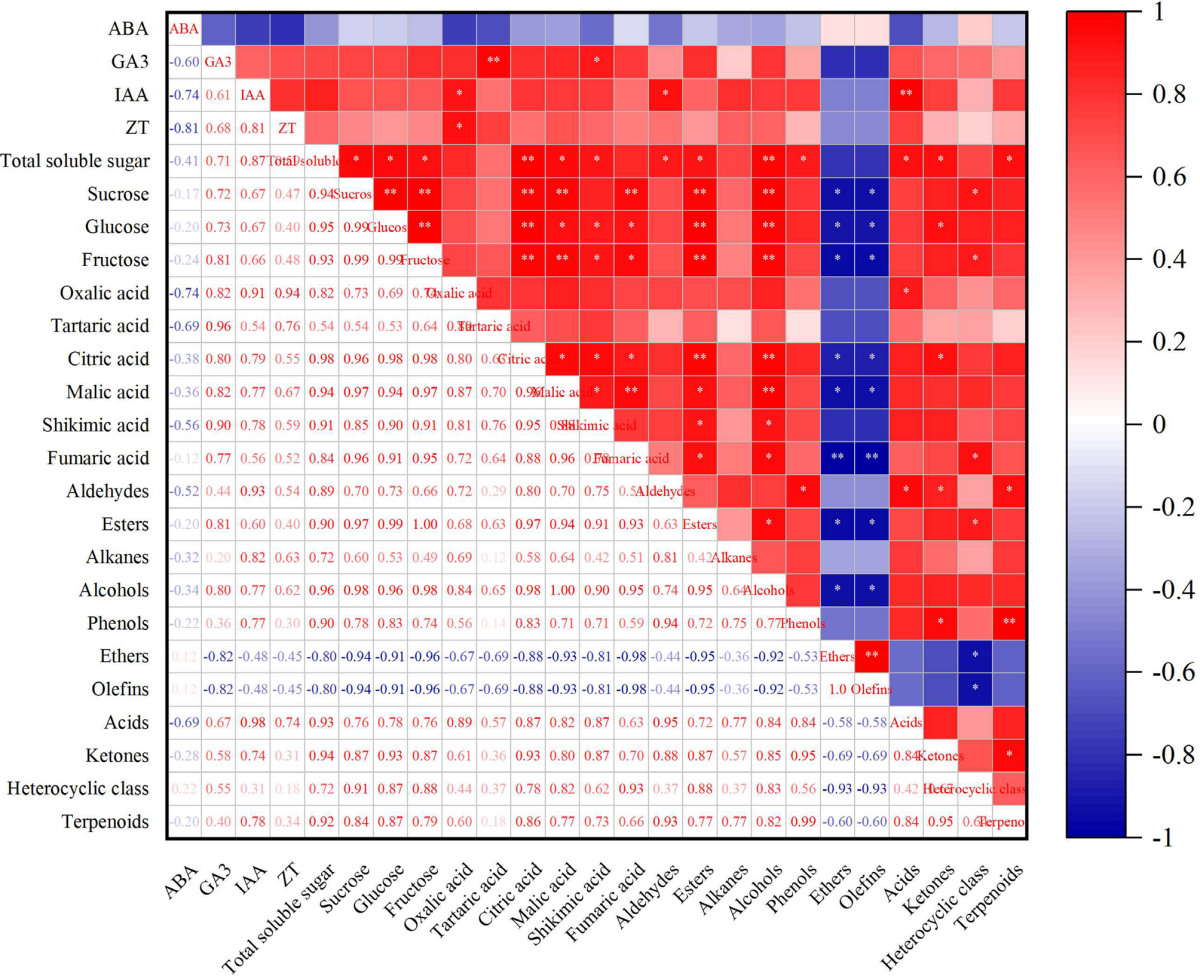
Cluster Heat Map of Volatile Aroma Components of ‘Chardonnay’ Fruit Treated with Exogenous Pro-Ca Notes: * means significant difference (*P* < 0.05), ** means extremely significant difference (*P* < 0.01)

The correlation between the contents of endogenous hormones, sugar and acid components and aroma substances in grape berries at 105 d after initial flowering (maturity stage) was analyzed by Origin software, and the reasons for the effect of Pro-Ca on fruit flavor quality were discussed. According to the correlation coefficient matrix (Fig. 7), there is a certain correlation between fruit endogenous hormones and the content of sugar and acid components and aroma substances, and there is also a certain correlation between the content of sugar and acid components and different aroma substances.

The correlation analysis between endogenous hormones and sugar-acid components and aroma substances in fruits showed that GA_3_ was significantly positively correlated with tartaric acid (*P* < 0.01) and shikimic acid (*P* < 0.05). IAA was significantly positively correlated with oxalic acid and aldehydes (*P* < 0.05), and was significantly positively correlated with acids (*P* < 0.01). ZT was positively correlated with oxalic acid (*P* < 0.05).

The results of correlation analysis between the content of sugar and acid components and different aroma substances showed that soluble sugar was significantly positively correlated with aldehydes, esters, phenols, acids, ketones and terpenes (*P* < 0.05), and extremely significantly positively correlated with alcohols (*P* < 0.01). Sucrose, glucose and fructose were significantly positively correlated with esters and alcohols (*P* < 0.01), and significantly negatively correlated with ethers and olefins (*P* < 0.05). Citric acid was significantly positively correlated with esters and alcohols (*P* < 0.01), significantly positively correlated with ketones (*P* < 0.05), and significantly negatively correlated with ethers and olefins (*P* < 0.05). Malic acid was significantly positively correlated with esters (*P* < 0.05), extremely significantly positively correlated with alcohols (*P* < 0.01), and significantly negatively correlated with ethers and olefins (*P* < 0.05).

### Correlation analysis between endogenous hormones, sugar and acid components and aroma substances in grape fruits

The contents of sugar, organic acid and aroma substances in ‘Chardonnay’ grape fruit were analyzed by principal component analysis at 105 d after the initial flowering (maturity stage). The results are shown in the following table. It can be seen from Table 2 that three principal components were extracted from ‘Chardonnay’ grapes, and the Eigen value of each principal component were greater than 1, and the cumulative variance contribution rate of these three principal components was 96.82%, indicating that the three principal components of wine grape ‘Chardonnay’ can reflect all the information of each index. Comprehensive evaluation of different concentrations of Pro-Ca treatment of ‘Chardonnay’ grapes, according to the principle of high score and good treatment effect, 600 mg·L^-1^ Pro-Ca treatment was the best. (Comprehensive score = variance contribution rate 1 × FAC1 + variance contribution rate 2 × FAC2 + variance contribution rate 3 × FAC3)

**Table 2:** The principal component score table of Pro-Ca treatment on ‘Chardonnay’ grapes

Treatment	FAC1	FAC2	FAC3	Comprehensive score	Ranking
0(Control)	-1.60	0.77	0.15	-1.18	5
200 mg · L-1Pro-Ca	-0.10	-0.97	-1.49	-0.28	4
400 mg · L-1Pro-Ca	0.61	0.46	0.06	0.54	2
600 mg · L-1Pro-Ca	1.02	0.92	-0.04	0.91	1
800 mg · L-1Pro-Ca	0.07	-1.18	1.32	0.01	3
Eigen value	16.73	2.31	1.29		
Variance contribution rate (%)	79.69	11.00	6.13		
Cumulative variance proportion (%)	79.69	90.69	96.82		

## Discussions

### The impact of pro-ca on endogenous hormones in ‘chardonnay’ grape berries

Plant endogenous hormones are trace organic substances found in plants that play a role in cell growth, division, and differentiation. They also regulate physiological processes such as seed dormancy, fruit development, tissue senescence, and stress resistance [24]. One of the significant inhibitory effects of ABA on plant growth during plant development is the acceleration of plant organ abscission, which contradicts the effects of IAA, GA, and ZT on plant growth [25].

The results demonstrated that, except for the 600 mg·L^-1^ Pro-Ca treatment, the ABA content in the fruits of the other treatments was higher at 45 d and 85 d after initial flowering. As the grape fruits approached maturity in the late growth stage, the ABA content decreased. This decrease can be attributed to the fact that ABA stimulated cell division and meristem activity in the young fruit stage and participated in the regulation of pigment synthesis and color change during the color conversion stage. In the later stage, the main processes of cell division and differentiation were completed as the fruit approached maturity, resulting in a decrease in ABA content. Additionally, ABA can regulate cell division and differentiation, as well as promote the synthesis of carotenoids, thus increasing the pigment content of plants [26, 27].

Furthermore, the application of 600 mg·L^-1^ Pro-Ca treatment significantly reduced the ABA content in the young fruit stage. However, at 65 d after initial flowering, the ABA content was higher compared to 45 d after initial flowering. At fruit ripening (105 d after initial flowering), there was no significant difference in fruit ABA content compared to the control, indicating that 600 mg·L^-1^ Pro-Ca treatment effectively promoted fruit ripening, as evidenced by the color change observed at 65 d after initial flowering. Previous studies have shown that Pro-Ca treatment can promote fruit ripening and reduce fruit drop in fruit trees, thereby increasing the fruit setting rate. This effect may be related to the impact of Pro-Ca on the endogenous ABA levels during the fruit setting period [28, 29].

The results also indicated that the GA_3_ content in grapes was highest at 45 d after initial flowering, while the IAA content was highest at the same interval. As the fruit ripened, the contents of GA_3_ and IAA gradually decreased. Research has revealed that seeds produce a substantial amount of gibberellin during the green fruit stage, which is then transported to the nearby short branches to inhibit the formation of flower buds and retain nutrients for the normal growth of the fruit [30]. During the fruit expansion period, the synthesis and transport activities of IAA were more active, promoting the elongation and expansion of fruit cells [31]. Previous studies have demonstrated that Pro-Ca can sustain the original activity level of gibberellin in plant tissues for an extended period of time [32]. The results displayed that Pro-Ca treatment significantly increased the contents of GA_3_ and IAA during fruit development. Garcia R et al. [33] discovered that hormone treatments such as GA_3_ increased fruit endogenous hormones, resulting in increased fruit yield and quality.

ZT is primarily stored in shoot tips, root tips, immature seeds, and growing fruits in plants. It possesses the ability to promote cell division, delay senescence, and prevent fruit drop [34]. The results revealed that the content of ZT was highest at 45 d after initial flowering, similar to the changing trend of GA_3_, and Pro-Ca treatment significantly increased the content of ZT.

In addition, the correlation analysis of endogenous hormones with sugar and acid components and aroma substances in mature fruits showed that GA_3_ was significantly positively correlated with tartaric acid (*P* < 0.01) and shikimic acid *(P* < 0.05). IAA was significantly positively correlated with oxalic acid and aldehydes (*P* < 0.05), and was significantly positively correlated with acids (*P* < 0.01). ZT was positively correlated with oxalic acid (*P* < 0.05). It shows that endogenous hormones in fruits can regulate fruit sugar and acid accumulation and fruit aroma substance content.

### The impact of pro-ca on the sugar- acid metabolism of ‘chardonnay’ grape berries

The sugar and organic acid content of grape berries is crucial for wine quality. Research indicates that in wine grapes, the majority of sugars are converted into alcohol, while approximately 10% is transformed into lipids and phenolic acids. Alterations in the types and quantities of organic acids have an impact on the taste, color, and stability of wine, as well as regulate the acid-base balance [35]. Grape fruits contain soluble sugars such as glucose, fructose, and sucrose, which play a crucial role in grape fruit quality. Studies have revealed a gradual increase in the content of glucose, fructose, and sucrose during the maturation and development of grape fruits. During the pre-ripening stage, grape metabolism primarily utilizes hexose, whereas post-veraison, hexose is predominantly accumulated [36]. The content of glucose, fructose, and sucrose exhibits an upward trend as the fruits near maturity, with fructose and glucose accumulating more prominently compared to sucrose. Furthermore, the application of Pro-Ca treatment can effectively enhance the content of soluble sugars in fruits, suggesting its ability to stimulate sugar accumulation, which is attributed to its promotion of plant photosynthesis. Increased photosynthesis benefits the accumulation of organic compounds [37].

Enzymes such as sucrose synthase(SuSy), sucrose phosphate synthase(SPS), acid invertase(AI), and neutral invertase(NI)mainly regulate the conversion and accumulation of sucrose, fructose, and glucose in plants [38]. SuSy performs a dual function by participating in both the synthesis and breakdown of sucrose. AI and NI catalyze the hydrolysis of sucrose into glucose and fructose, whereas SPS facilitates the conversion of fructose and glucose into sucrose [39]. The conversion direction of SuSy relies on its phosphorylation state and is traditionally associated with its involvement in sucrose breakdown [40]. However, SuSy may also play a significant role in sucrose synthesis in photosynthetic organs, which can vary among different plant species [41]. Sucrose is a vital product of plant photosynthesis and serves as a critical means for long-distance carbohydrate transport [42]. Research indicates that SuSy and SPS are the primary factors contributing to sucrose synthesis in grapes, whereas AI and NI are the key enzymes influencing fruit sweetness [43]. During fruit growth and maturation, sucrose synthase synthesis(SuSy-s)activity declines, SPS activity remains relatively stable, but decreases during the ripening stage. NI and sucrose synthase cleavage(SuSy-c)activities gradually decrease, while AI activity increases as the fruit develops, potentially linked to the accumulation of fructose and glucose in ripe fruit. Throughout fruit ripening, AI and NI display heightened activity, facilitating the buildup of fructose and glucose in the fruit [44].

Organic acids play a crucial role in plants, particularly in determining the taste, aroma, and overall quality of fruits [45]. Grape fruits primarily contain tartaric acid and malic acid as the primary organic acids [46].Malic acid is vital for fruit development, and its concentration significantly influences the ultimate quality of the fruit [47].Malic acid is closely associated with the conversion of sugars during grape fruit ripening, serving as a carbon source for sugar accumulation [48].Ripe grape fruits also contain a significant amount of tartaric acid, while the levels of citric acid and oxalic acid are relatively low [47].Experimental findings indicate a gradual decrease in the levels of tartaric acid, malic acid, and oxalic acid as the fruit matures, alongside a corresponding slow increase in citric acid content. As the fruit approaches ripening, the order of organic acid content is as follows: tartaric acid > malic acid > citric acid > oxalic acid. The application of Pro-Ca treatment can effectively enhance the content of tartaric acid, malic acid, citric acid, and oxalic acid in grape fruits, particularly during the period when the fruit is approaching ripeness. This suggests that Pro-Ca treatment assists in facilitating the buildup of organic acids in grape fruits. The accumulation of different organic acids in fruits relies on the equilibrium between their synthesis, breakdown, and utilization [49]. Tartaric acid, malic acid, succinic acid, oxalic acid, and citric acid are the primary organic acids that influence the flavor of grape fruits. They are directly or indirectly regulated by key enzymes such as phosphoenolpyruvate carboxylase(PEPC), NADP-malate enzyme(NADP-ME), NAD-isocitrate dehydrogenase(NADP-IDH), cytoplasmic aconitic acid enzyme(Cyt-ACO), NAD-malate dehydrogenase(NAD-MDH), and citrate acid synthase(CS) [50]. Specifically, enzymes like NAD-MDH and PEPC control the synthesis of malic acid, whereas NADP-ME regulates its degradation [51]. During the ripening period, which occurs 105 d after the initial flowering, the activities of key enzymes NAD-MDH and PEPC decrease. These enzymes play a crucial role in controlling the synthesis of malic acid, and their reduced activity results in a decrease in malic acid synthesis. Conversely, the activity of NADP-ME, the enzyme responsible for malic acid degradation, remains steady throughout the ripening period. This suggests that the degradation rate of malic acid in the fruit does not change significantly. Citric acid is synthesized through the production of oxaloacetic acid and the action of citrate synthase [52]. Experimental results demonstrate that the activities of CS and PEPC gradually decrease as the fruit ripens. This decline is associated with CS’s role in regulating the acid-base balance and enhancing the fruit’s sweetness and aroma [53]. Previous studies have indicated that Pro-Ca can influence the acid-sugar balance in fruits by regulating source-sink balance and nutrient allocation [28]. The findings of this study demonstrate that the application of 600 mg·L^-1^ Pro-Ca treatment can substantially boost the activity of enzymes like PEPC, NADP-ME, NADP-IDH, Cyt-ACO, NAD-MDH, and CS. This enhancement is advantageous for the accumulation of organic acids in fruits and the enhancement of grape fruit quality.

The correlation analysis between the content of sugar and acid components and aroma substances showed that there was a certain correlation between the content of sugar and acid components and different aroma substances. Soluble sugar was significantly positively correlated with aldehydes, esters, phenols, acids, ketones and terpenes (*P* < 0.05), and extremely significantly positively correlated with alcohols (*P* < 0.01). Sucrose, glucose and fructose were significantly positively correlated with esters and alcohols (*P* < 0.01), and significantly negatively correlated with ethers and olefins (*P* < 0.05). Citric acid was significantly positively correlated with esters and alcohols (*P* < 0.01), significantly positively correlated with ketones (*P* < 0.05), and significantly negatively correlated with ethers and olefins (*P* < 0.05). Malic acid was significantly positively correlated with esters (*P* < 0.05), extremely significantly positively correlated with alcohols (*P* < 0.01), and significantly negatively correlated with ethers and olefins (*P* < 0.05). The study of Fan X et al. [54] also showed that the synthesis of fruit aroma substances was related to fruit sugar and organic acids. Therefore, increasing the sugar and acid content of wine grape fruit plays an important role in increasing the content of fruit aroma substances, which can provide better raw materials for wine making.

### The impact of pro-ca on the volatile aroma compounds of ‘chardonnay’ grape berries

The GC-MS analysis detected a total of 36 volatile aroma substances in this experiment. Varying concentrations of Pro-Ca treatment led to variations in both the types and concentrations of the detected aroma substances. Alcohol compounds are metabolic byproducts resulting from the decomposition of sugars, decarboxylation, and deamination of amino acids [55].Alcohol compounds produce a distinct “heteroalcohol” aroma in red wine when their concentration exceeds 400 mg·L^-1^; at concentrations lower than this threshold, they contribute to a more “complex” aroma [56].The experimental findings indicate that Pro-Ca treatment enhances the diversity of alcohol compounds in ‘Chardonnay’ grapes and significantly boosts the overall alcohol content. Ester compounds are formed through the condensation of alcohols and carboxylic acids. They belong to a group of volatile aromatic compounds that constitute the primary components of wine aroma and play a vital role in the winemaking process. Moreover, they are the key contributing factors to its flavor. Typically, small and medium-chain esters exhibit a “fruity” aroma, while large-chain esters possess a “soapy” characteristic [57]. Seven ester compounds were identified in the wine grapes during this experiment, and Pro-Ca treatment resulted in an increase in both the variety and quantity of lipid compounds. Notably, Diethyl Phthalate and Formic acid, ethenyl ester exhibited higher concentrations among them. This observation aligns with the general presence of ethyl acetate (resulting from the condensation of ethanol and fatty acid) or ethyl ester (arising from the condensation of ethanol and acetic acid) in wines [56]. To summarize, the application of Pro-Ca treatment affects the aroma profile of ‘Chardonnay’ grapes, leading to an increase in both the diversity and concentration of alcohol and ester compounds. These findings imply that Pro-Ca treatment could potentially enhance the flavor and aroma characteristics of grapes.

Aldehyde and ketone compounds are a type of compounds that contribute to the fruity aroma and can be formed through the oxidation of alcohols. Nevertheless, these compounds are prone to instability and subsequent oxidation into carboxylic acids, resulting in a reduction of their concentration [58]. During Pro-Ca treatment, while there is a decrease in the variety of aldehyde and ketone compounds, their content significantly rises in each treatment group compared to the control group, which is advantageous for the winemaking process. Terpenoids are commonly found in fruits as glycosides. In the process of winemaking, they undergo hydrolysis by acids or enzymes to generate free volatile compounds, imparting a fruity and floral aroma to the wine. Terpenoids are crucial flavor components in wine, possessing a low aroma threshold, whereby even at low concentrations, they make a substantial contribution to the overall aroma of the wine [59]. This experiment identified solely one terpenoid compound, namely Cyclohexanol, 5-methyl-2-(1-methylethyl)-, in the wine grapes; however, Pro-Ca treatment led to an elevation in the terpenoid content within the grapes. Phenolic substances contribute significantly to the characteristics of wine, influencing its appearance, astringency, taste, health benefits, and serving as a key factor in the diversity of wine flavors [60]. This experiment identified three types of phenolic substances in wine grapes, namely Phenol, 4-ethyl-2-methoxy-, and Eugenol, with exclusive detection in the Pro-Ca treatment group. Notably, in the Pro-Ca treatment group with a concentration of 600 mg·L^-1^, the content of phenolic substances reached the highest level, surpassing other treatment groups significantly.

Based on a comprehensive evaluation of the effects of Pro-Ca treatment on the quality of wine grape fruit, the most optimal concentration for foliar spraying is determined to be 600 mg·L^-1^ Pro-Ca. This conclusion is drawn from the principal component analysis of the sugar, organic acid, and aroma substance content of ‘Chardonnay’ grape fruit at 105 d after initial flowering (maturity stage).

## Conclusions

The results demonstrate the effectiveness of Pro-Ca in reducing ABA content during fruit development and increasing the levels of ZT, GA_3_, and IAA. This treatment promotes fruit ripening and enhances the activities of enzymes involved in sugar and organic acid synthesis, leading to higher sugar and organic acid content in the fruit. Moreover, Pro-Ca treatment improves both the variety and amount of fruit aroma substances, with the best effect observed at a concentration of 600 mg·L^− 1^. Principal component analysis confirms that 600 mg·L^− 1^ Pro-Ca is the optimal concentration for foliar spraying.

## Data Availability

No datasets were generated or analysed during the current study.

## References

[1] Borsani J, Budde CO, Porrini L, Lauxmann MA, Lombardo VA, Murray R, Andreo CS, Drincovich MF, Lara MV (2009). Carbon metabolism of peach fruit after harvest: changes in enzymes involved in organic acid and sugar level modifications. J Exp Bot.

[2] Sweetman C, Deluc LG, Cramer GR, Ford CM, Soole KL (2009). Regulation of malate metabolism in grape berry and other developing fruits. Phytochemistry.

[3] Wang Q, Gao F, Chen X, Wu W, Wang L, Shi J, Huang Y, Shen Y, Wu G, Guo J (2022). Characterization of key aroma compounds and regulation mechanism of aroma formation in local Binzi (Malus pumila × Malus asiatica) fruit. BMC Plant Biol.

[4] Cao X, Wei C, Duan W, Gao Y, Kuang J, Liu M, Chen K, Klee H, Zhang B (2021). Transcriptional and epigenetic analysis reveals that NAC transcription factors regulate fruit flavor ester biosynthesis. Plant J.

[5] Giagnoni L, Maienza A, Baronti S, Vaccari FP, Genesio L, Taiti C, Martellini T, Scodellini R, Cincinelli A, Costa C, Mancuso S, Renella G (2019). Long-term soil biological fertility, volatile organic compounds and chemical properties in a vineyard soil after biochar amendment. Geoderma.

[6] Wei M, Ma T, Ge Q, Li C, Zhang K, Fang Y, Sun X (2022). Challenges and opportunities of winter vine pruning for global grape and wine industries. J Clean Prod.

[7] Doolette C, Read T, Howell N, Cresswell T, Lombi E (2020). Zinc from foliar-applied nanoparticle fertiliser is translocated to wheat grain: a 65Zn radiolabelled translocation study comparing conventional and novel foliar fertilisers. Sci Total Environ.

[8] Quamruzzaman M, Manik SMN, Shabala S, Zhou M. Improving Performance of Salt-Grown Crops by Exogenous Application of Plant Growth Regulators. Biomolecules 2021, 11, 788. 10.3390/biom11060788 PMC8225067 34073871

[9] Ferrari AM, Pini M, Sassi D, Zerazion E, Neri P (2018). Effects of grape quality on the environmental profile of an Italian vineyard for Lambrusco red wine production. J Clean Prod.

[10] Paulson GS, Hull LA, Biddinger DJ (2005). Effect of a Plant Growth Regulator Prohexadione-Calcium on Insect pests of Apple and Pear. J Econ Entomol.

[11] Chang P (2016). Influence of Prohexadione-Calcium on the growth and quality of summer ‘Jen-Ju Bar’ Guava Fruit. J Plant Growth Regul.

[12] Lal M, Mir M, Waida UI, Kumar A (2018). Response of prohexadione calcium and paclobutrazol on growth and physio-chemical characteristics of pear cv. Clapp’s favorite. Indian J Hortic.

[13] Roux C, Lemarquand A, Orain G, Campion C, Simoneau P, Poupard P (2006). Effects of the plant growth regulator prohexadione-calcium and the SAR-inducer acibenzolar-S-methyl on the quality of apples at harvest. J Hortic Sci Biotechnol.

[14] Gonzalo-diago A, Avizcuri J, Ortigosa N, Dizy M, Martinez-soria M, Sanz-asensio J, Echavarri F, Fernández P (2012). Prohexadione-calcium as a regulator of vine growth: Effect on the physical-chemical characteristics of wine grapes.

[15] Avizcuri J, Gonzalo-diago A, Sanz-asensio J, Martinez-soria M, López-alonso M, Dizy M, Echavarri F, Vaquero-fernández L, Fernández P (2013). Effect of cluster thinning and prohexadione calcium applications on phenolic composition and sensory properties of red wines. J Agric Food Chem.

[16] De oliveira LS, Soratto RP, Cairo PAR, Da silva LD, Matsumoto SN, Silva RDA (2022). Common Bean Plant size and yield in response to Rates of Foliar-Applied Paclobutrazol, Mepiquat Chloride, and Prohexadione Calcium. J Plant Growth Regul.

[17] Chi Z, Wang Y, Deng Q, Zhang H, Pan C, Yang Z (2020). Endogenous phytohormones and the expression of flowering genes synergistically induce flowering in loquat. J Integr Agric.

[18] Dien DC, Mochizuki T, Yamakawa T (2019). Effect of various drought stresses and subsequent recovery on proline, total soluble sugar and starch metabolisms in Rice varieties. Plant Prod Sci.

[19] Wang S, Jin N, Jin L, Xiao X, Hu L, Liu Z, Wu Y, Xie Y, Zhu W, Lyu J, Yu J. Response of tomato fruit quality depends on period of led supplementary light. Front Nutr 2022, 9. 10.3389/fnut.2022.833723 PMC8841748 35174200

[20] Joshi SS, Datir SS, Pawar MW, Nerkar YS (2006). Sucrose metabolism in different sugar beet cultivars. Sugar Tech.

[21] Ma B, Yuan Y, Gao M, Li C, Ogutu C, Li M, Ma F (2018). Determination of predominant organic acid components in malus species: correlation with apple domestication. Metabolites.

[22] Tang M, Bie Z, Wu M, Yi H, Feng J (2010). Changes in organic acids and acid metabolism enzymes in melon fruit during development. Sci Hort.

[23] Wu Y, Duan S, Zhao L, Gao Z, Luo M, Song S, Xu W, Zhang C, Ma C, Wang S (2016). Aroma characterization based on aromatic series analysis in table grapes. Sci Rep.

[24] Kumar R, Khurana A, Sharma AK (2014). Role of plant hormones and their interplay in development and ripening of fleshy fruits. J Exp Bot.

[25] Li J, Liu B, Li X, Li D, Han J, Zhang Y, Ma C, Xu W, Wang L, Jiu S, Zhang C, Wang S (2021). Exogenous abscisic acid mediates berry quality improvement by altered endogenous plant hormones level in ruiduhongyu grapevine. Front Plant Sci.

[26] Lee S, Jang S, Yoon E, Heo J, Chang K, Choi J, Dhar S, Kim G, Choe J, Heo J, Kwon C, Ko J, Hwang Y, Lim J (2016). Interplay between ABA and GA modulates the timing of asymmetric cell divisions in the Arabidopsis root ground tissue. Mol Plant.

[27] Li Z, Ahammed GJ (2023). Hormonal regulation of anthocyanin biosynthesis for improved stress tolerance in plants. Plant Physiol Biochem.

[28] Musacchi S, Sheick R, Mia MJ, Serra S (2023). Studies on physiological and productive effects of multi-leader training systems and Prohexadione-Ca applications on apple cultivar ‘WA 38′. Scientia Hortic.

[29] Greene DW (2008). The effect of repeat annual applications of prohexadione–calcium on fruit set, return bloom, and fruit size of apples. HortScience Horts.

[30] Megan T, Carl-Erik T, Zhao X, Grabowski P, Doerge R, Ma J, Volenec J, Evans J, Ramstein GP, Buell CR, Casler MD, Jiang. Y. Genome-wide association study in pseudo-f2 populations of switchgrass identifies genetic loci affecting heading and anthesis dates. Front Plant Sci 2018, 9. 10.3389/fpls.2018.01250 PMC6146286 30271414

[31] Zhang Y, Yan Y, Fu C, Li M, Wang Y (2016). Zinc sulfate spray increases activity of carbohydrate metabolic enzymes and regulates endogenous hormone levels in apple fruit. Sci Hort.

[32] W R (2000). Growth retardants: effects on gibberellin biosynthesis and other metabolic pathways [Review]. Annu Rev Plant Physiol Plant Mol Biol.

[33] Garcia R, Siebeneichler S, Oliveira M, Santos E, Adorian G, Lorençoni R, Alves veloso R, Souza C (2019). Application of GA_3_ and harvest season interfere in pineapple yield and fruit quality. Nucleus.

[34] Sosnowski J, Truba M, Vasileva V (2023). The impact of auxin and cytokinin on the growth and development of selected crops. Agriculture.

[35] Li S, Duan C, Han Z (2023). Grape polysaccharides: compositional changes in grapes and wines, possible effects on wine organoleptic properties, and practical control during winemaking. Crit Rev Food Sci Nutr.

[36] Jia H, Xie Z, Wang C, Shangguan L, Qian N, Cui M, Liu Z, Zheng T, Wang M, Fang J (2017). Abscisic acid, sucrose, and auxin coordinately regulate berry ripening process of the Fujiminori grape. Funct Integr Genom.

[37] Li Y, Zhou H, Feng N, Zheng D, Ma G, Feng S, Liu M, Yu M, Huang X, Huang A (2023). Physiological and transcriptome analysis reveals that prohexadione-calcium promotes rice seedling’s development under salt stress by regulating antioxidant processes and photosynthesis. PLoS ONE.

[38] Umer MJ, Bin safdar L, Gebremeskel H, Zhao S, Yuan P, Zhu H, Kaseb MO, Anees M, Lu X, He N, Gong C, Liu W (2020). Identification of key gene networks controlling organic acid and sugar metabolism during watermelon fruit development by integrating metabolic phenotypes and gene expression profiles. Hortic Res.

[39] Wang S, Pan C, Zhang C, Zhao S (2023). Key enzymes of sucrose metabolism play an important role in source–sink regulation of walnut fruit growth and development. New Z J Crop Hortic Sci.

[40] Tanase K, Shiratake K, Mori H, Yamaki S (2002). Changes in the phosphorylation state of sucrose synthase during development of Japanese pear fruit. Physiol Plant.

[41] Moriguchi T, Abe K, Sanada T, Yamaki S (1992). Levels and role of sucrose synthase, sucrose-phosphate synthase, and acid invertase in sucrose accumulation in fruit of Asian pear. Am Soc Hortic Sci.

[42] Pelah D, Wang W, Altman A, Shoseyov O, Bartels D (2006). Differential accumulation of water stress-related proteins, sucrose synthase and soluble sugars in Populus species that differ in their water stress response. Physiol Plant.

[43] Zhu S, Liang Y, An X, Kong F, Gao D, Yin H (2017). Changes in sugar content and related enzyme activities in table grape (Vitis vinifera L.) in response to foliar selenium fertilizer. J Sci Food Agric.

[44] Tan S, Xie J, Wang W, Shi S (2019). Effects of exogenous plant hormones on sugar accumulation and related enzyme activities during the development of longan (Dimocarpus Longan Lour.) Fruits. J Hortic Sci Biotechnol.

[45] Jiang B, Fang X, Fu D, Wu W, Han Y, Chen H, Liu R, Gao H (2022). Exogenous salicylic acid regulates organic acids metabolism in postharvest blueberry fruit. Front Plant Sci.

[46] Cholet C, Claverol S, Claisse O, Rabot A, Osowsky A, Dumot V, Ferrari G (2016). Tartaric acid pathways in Vitis vinifera L. (Cv. Ugni Blanc): a comparative study of two vintages with contrasted climatic conditions. BMC Plant Biol.

[47] Sheng J, Baldeck JD, Nguyen PT, Quivey RG, Marquis RE (2010). Alkali production associated with malolactic fermentation by oral streptococci and protection against acid, oxidative, or starvation damage. Can J Microbiol.

[48] Zhou Y, He W, Zheng W, Tan Q, Xie Z, Zheng C, Hu C (2018). Fruit sugar and organic acid were significantly related to fruit mg of six citrus cultivars. Food Chem.

[49] Yang C, Chen T, Shen B, Sun S, Song H, Chen D, Xi W (2019). Citric acid treatment reduces decay and maintains the postharvest quality of peach (Prunus persica L.) fruit. Food Sci Nutr.

[50] Zhu J, Li C, Fan Y, Qu L, Huang R, Liu J, Zhang C, Ge Y (2022). γ-Aminobutyric acid regulates mitochondrial energy metabolism and organic acids metabolism in apples during postharvest ripening. Postharvest Biol Technol.

[51] Chen F, Liu X, Chen L (2009). Developmental changes in pulp organic acid concentration and activities of acid-metabolising enzymes during the fruit development of two loquat (Eriobotrya japonica Lindl.) Cultivars differing in fruit acidity. Food Chem.

[52] Davies JN, Maw GA (1972). Metabolism of citric and malic acids during ripening of tomato fruit. J Sci Food Agric.

[53] Liu J, Chi G, Jia C, Zhang J, Xu B, Jin Z (2013). Function of a citrate synthase gene (MaGCS) during postharvest banana fruit ripening. Postharvest Biol Technol.

[54] Fan X, Lu N, Xu W, Zhuang Y, Jin J, Mao X, Ren N. Response of Flavor substances in Tomato Fruit to Light Spectrum and Daily Light Integral. Volume 12. Plants; 2023. p. 2832. 10.3390/plants12152832 PMC10420795 37570986

[55] Zhang S, Petersen MA, Liu J, Toldam-andersen TB (2015). Influence of Pre-fermentation treatments on wine volatile and sensory Profile of the New Disease Tolerant Cultivar Solaris. Molecules.

[56] Longo R, Carew A, Sawyer S, Kemp B, Kerslake F (2021). A review on the aroma composition of Vitis vinifera L. Pinot noir wines: origins and influencing factors. Crit Rev Food Sci Nutr.

[57] Adlard ER, Jokie Bakker, Ronald J, Clarke. Wine Flavour Chemistry (2nd Edn). Chromatographia 2018, 81, 1241–1241.

[58] Petropulos VI, Bogeva E, Stafilov T, Stefova M, Siegmund B, Pabi N, Lankmayr E (2014). Study of the influence of maceration time and oenological practices on the aroma profile of Vranec wines. Food Chem.

[59] Cai J, Zhu B, Wang Y, Lu L, Lan Y, Reeves MJ, Duan (2014). C. Influence of pre-fermentation cold maceration treatment on aroma compounds of Cabernet Sauvignon wines fermented in different industrial scale fermenters. Food Chem.

[60] Gawel R, Smith PA, Waters EJ (2016). Influence of polysaccharides on the taste and mouthfeel of white wine. Aust J Grape Wine Res.

